# Repertoire of novel sequence signatures for the detection of *Candidatus* Liberibacter asiaticus by quantitative real-time PCR

**DOI:** 10.1186/1471-2180-14-39

**Published:** 2014-02-17

**Authors:** Sunitha Kogenaru, Qing Yan, Nadia Riera, M Caroline Roper, Xiaoling Deng, Timothy A Ebert, Michael Rogers, Michael E Irey, Gerhard Pietersen, Charles M Rush, Nian Wang

**Affiliations:** 1 Citrus Research and Education Center, Department of Microbiology and Cell Science, IFAS, University of Florida, Lake Alfred 33850, USA; 2 Present address: Division of Nephrology, Department of Internal Medicine, University of Michigan Medical School, Ann Arbor, MI 48109-0676, USA; 3 Department of Plant Pathology and Microbiology, University of California, Riverside, CA 92521, USA; 4 Department of Plant Pathology, South China Agricultural University, Guangzhou, Guangdong, P. R. China; 5 Department of Entomology and Nematology, Citrus Research and Education Center, IFAS, University of Florida, Lake Alfred 33850, USA; 6 US Sugar Corporation, Clewiston, FL 33440, USA; 7 Department of Microbiology & Plant Pathology, ARC-Plant Protection Research Institute, University of Pretoria, Pretoria, South Africa; 8 Texas A&M AgriLife Research and Extension Center, Texas A&M University, Amarillo, USA

**Keywords:** Detection system, Diagnostic, *Candidatus* Liberibacter asiaticus, Greening, Huanglongbing, Bacteria, Psyllid, Citrus

## Abstract

**Background:**

Huanglongbing (HLB) or citrus greening is a devastating disease of citrus. The gram-negative bacterium *Candidatus* Liberibacter asiaticus (Las) belonging to the α-proteobacteria is responsible for HLB in North America as well as in Asia. Currently, there is no cure for this disease. Early detection and quarantine of Las-infected trees are important management strategies used to prevent HLB from invading HLB-free citrus producing regions. Quantitative real-time PCR (qRT-PCR) based molecular diagnostic assays have been routinely used in the detection and diagnosis of Las. The oligonucleotide primer pairs based on conserved genes or regions, which include 16S rDNA and the β-operon, have been widely employed in the detection of Las by qRT-PCR. The availability of whole genome sequence of Las now allows the design of primers beyond the conserved regions for the detection of Las explicitly.

**Results:**

We took a complimentary approach by systematically screening the genes in a genome-wide fashion, to identify the unique signatures that are only present in Las by an exhaustive sequence based similarity search against the nucleotide sequence database. Our search resulted in 34 probable unique signatures. Furthermore, by designing the primer pair specific to the identified signatures, we showed that most of our primer sets are able to detect Las from the infected plant and psyllid materials collected from the USA and China by qRT-PCR. Overall, 18 primer pairs of the 34 are found to be highly specific to Las with no cross reactivity to the closely related species *Ca*. L. americanus (Lam) and *Ca.* L. africanus (Laf).

**Conclusions:**

We have designed qRT-PCR primers based on Las specific genes. Among them, 18 are suitable for the detection of Las from Las-infected plant and psyllid samples. The repertoire of primers that we have developed and characterized in this study enhanced the qRT-PCR based molecular diagnosis of HLB.

## Background

Huanglongbing (HLB) or citrus greening is the most devastating disease of citrus, threatening the citrus industry worldwide, and leading to massive reduction in fruit production as well as death of infected trees [1]. The causal agents of HLB are three closely related gram-negative, phloem-limited α-proteobacteria *Candidatus* Liberibacter species [2,3]. The heat tolerant strain *Ca.* L. asiaticus (Las) is the most widespread in Asia as well as in the USA whereas *Ca.* L. americanus (Lam) is mostly limited to South America [2-4]. *Ca.* L. africanus (Laf) is heat sensitive and localized to the African continent. All the three Liberibacter species are currently uncultured and are known to reside in the sieve tubes of the plant phloem [5] or in the gut of the phloem-feeding psyllids [6]. Psyllids are the natural vectors in transmitting the bacteria between plants [1,6]. The Asian psyllid, *Diaphorina citri* Kuwayama (Homoptera: Psyllidae) is responsible for transmitting Las and Lam in Asia and America, while the African citrus psyllid, *Trioza erytreae* Del Guercio (Homoptera: Psyllidae), is the natural vector of Laf in Africa [7]. The characteristic symptoms of the infected plants include the yellow shoots, foliar blotchy mottles, along with poor flowering and stunting [1]. HLB also results in poorly colored, unpleasant tasting, reduced size fruit that shows staining of vascular columella and seed abortion [1]. Generally the fruit may remain partially green, for this reason HLB is also called citrus greening [1]. Chronically infected trees are sparsely foliated and display extensive twig or limb die-back and eventually die within three to five years [1]. Moreover, the disorders induced in diseased plants vary with cultivar, tree maturity, time of infection, stages of disease and other abiotic or biotic agents that affect the tree [1]. HLB symptoms also share certain similarities to nutrient deficiency [1], citrus stubborn disease caused by *Spiroplasma citri*[8] and a HLB-like disease caused by a phytoplasma [9,10]. Early diagnosis and differentiation of Las infections from those defects and agents mentioned above, is thus critical to reducing the spread and devastation of this disease locally and via international trade, as well as minimizing the economic impact of potential false positive diagnoses.

Importantly, HLB and the Asian citrus psyllid (*D. citri*) are expanding to new citrus production areas. Currently, Asian citrus psyllid has been found in Florida, Texas, California, Arizona, Hawaii, Louisiana, Georgia, and Alabama in the USA, as well as in parts of South and Central America, Mexico, and the Caribbean. Meanwhile, HLB has not only been identified in Florida, Louisiana, South Carolina, Louisiana, Georgia, Texas and California of the USA; it has also been discovered in Cuba, Belize, Jamaica, Mexico, and other countries in the Caribbean [11]. While HLB and *D. citri* have been found in different producing areas, the number of infected trees and the psyllid vector population vary dramatically among different regions. Thus, different strategies of management of HLB are recommended for different regions, according to the corresponding severity of HLB and occurrence of psyllid vectors.

Currently, no efficient management strategy is available to control HLB. For the recently Las-infected citrus producing areas such as California, prevention and eradication of HLB are the most efficient and cost-effective approaches. Additionally, Las infected trees are most often found to be asymptomatic during the early stage of infection. Thus, accurate early detection of Las in citrus plants and psyllids is critical for enacting containment measures in non-endemic citrus producing areas. For the citrus producing areas without HLB, such as the Mediterranean region, accurate detection is critical for the success of quarantine measures against *Ca*. Liberibacter.

Methods such as biological indexing using graft, dodder transmission [12], isothermal loop amplification (LAMP) [13], electron microscopy [1], DNA probes [14], enzyme-linked immunosorbent assays (ELISA) [15], conventional PCR [16-22] and quantitative real-time PCR (qRT-PCR) [22-26] are used for the diagnosis and confirmation of HLB. Although diagnostic tools like conventional PCR and LAMP showed good sensitivity, they were not consistent in detection of Las bacterium from infected plant and psyllid materials [6,13,25]. The current HLB diagnostic detection mainly employs qRT-PCR based methods due to their sensitive and quantitative nature. The initial qRT-PCR oligonucleotide primer sets for the detection of Las, targeted *rplKAJL-rpoBC* operon (β-operon: CQULA04f/r) [26], 16S ribosomal RNA gene (rDNA) (HLBasf/r) [23], EUB338f/EUB518r [27], ALF518f/ EUB518r [27] or species specific variable regions. EUB338f/EUB518r primers are universal to Eubacteria [27], while ALF518f/EUB518r primers identify α-proteobacteria universally [27] including Las, therefore not specific. Furthermore, the primers based on the conserved 16S and β-operon regions are popular but nevertheless have been shown to pose a potential specificity issue, as both false negatives and false positives have been reported [28]. Therefore, efforts have been directed towards developing effective qRT-PCR primers that target other non-conserved sequences. Recent studies made use of intragenic repeat regions of the prophage sequence for the detection of Las by qRT-PCR [25]. However, the intragenic repeat regions of the prophage sequence were also identified in Lam. Therefore, these primer pairs, hyvi/hyvii did not distinguish between Las and Lam, posing a specificity issue [25]. Consequently, primer pairs that specifically detect Las and make clear distinction among other phylogenetically closely related bacteria are essential.

Here we took a complimentary approach to identify the genes that are unique to Las by a bioinformatic analysis with the goal of expanding the arsenal of tools for Las detection. The advancement in the genome sequencing of Las [29] provides an opportunity to design primers based on species specific sequences for the detection of Las. We designed the oligonucleotide primer pairs specific to the identified unique genic signatures. We further validated their specificities and selectivity against closely related strains that demonstrated the application to Las-infected tissues and insect vectors by a qRT-PCR.

## Results and discussion

Recently, the whole genome sequences of Las [29,30] have been sequenced. This allows for systematic screening of unique Las genes in a genome-wide fashion. The availability of the genome sequences of the closely related species Lam [31], *L. crescens* (Lcr) [32] and *Ca. L. solanacearum* (Lso) [33], further effectively helps in identification of unique regions, by minimizing the cross-species reactions, thereby enhancing the diagnostic identification of Las in a more distinct manner.

### Bioinformatic analysis

Several high-throughput applications have been developed recently to design diagnostic primers using the whole genome sequence information including KPATH, Insignia, TOFI, and TOPSI [34-40]. Among them, KPATH, Insignia, and TOPSI have the potential to be used for design of real-time PCR primers for qRT-PCR based assays for Las, whereas TOFI is used to design signatures for microarray-based assays. These methods mentioned above can be basically categorized into alignment-free and alignment-based approaches. The alignment-free approach uses both coding and non-coding regions of the genome and is useful for the genomes with less accurate sequence information, but generally result in high false positive rates as it does not involve pre-screening of the selected genomic loci for their discriminatory ability [37]. The alignment-based approach involves pre-screening of the selected genomic loci for their discriminatory ability [34]. This approach does not consider the genome annotation of genic and non-genic information, but rather aligns bigger regions of the genome, hence prone to lose shorter discriminatory sequence regions. Additionally, discriminatory ability of the selected regions are screened bioinformatically only on limited number of closely related species, which provide more opportunities for false positives. We therefore took a complementary bioinformatics approach by pre-screening shorter genic regions against the nucleotide sequence database (nt) at NCBI, to identify all the possible unique genic regions from the Las genome. The natural selection acts more strongly on genic region, hence use of discriminatory sequences in this region results in less false positives as the organisms are under selection pressure [41]. Additionally, pre-screening against the nt is more effective as it contains the largest pool of well-annotated nucleotide sequences from different organisms. We envisioned that these two steps would result in more specific detection of target organism with less false positives, hence are included in our bioinformatics approach.

**Figure 1:**
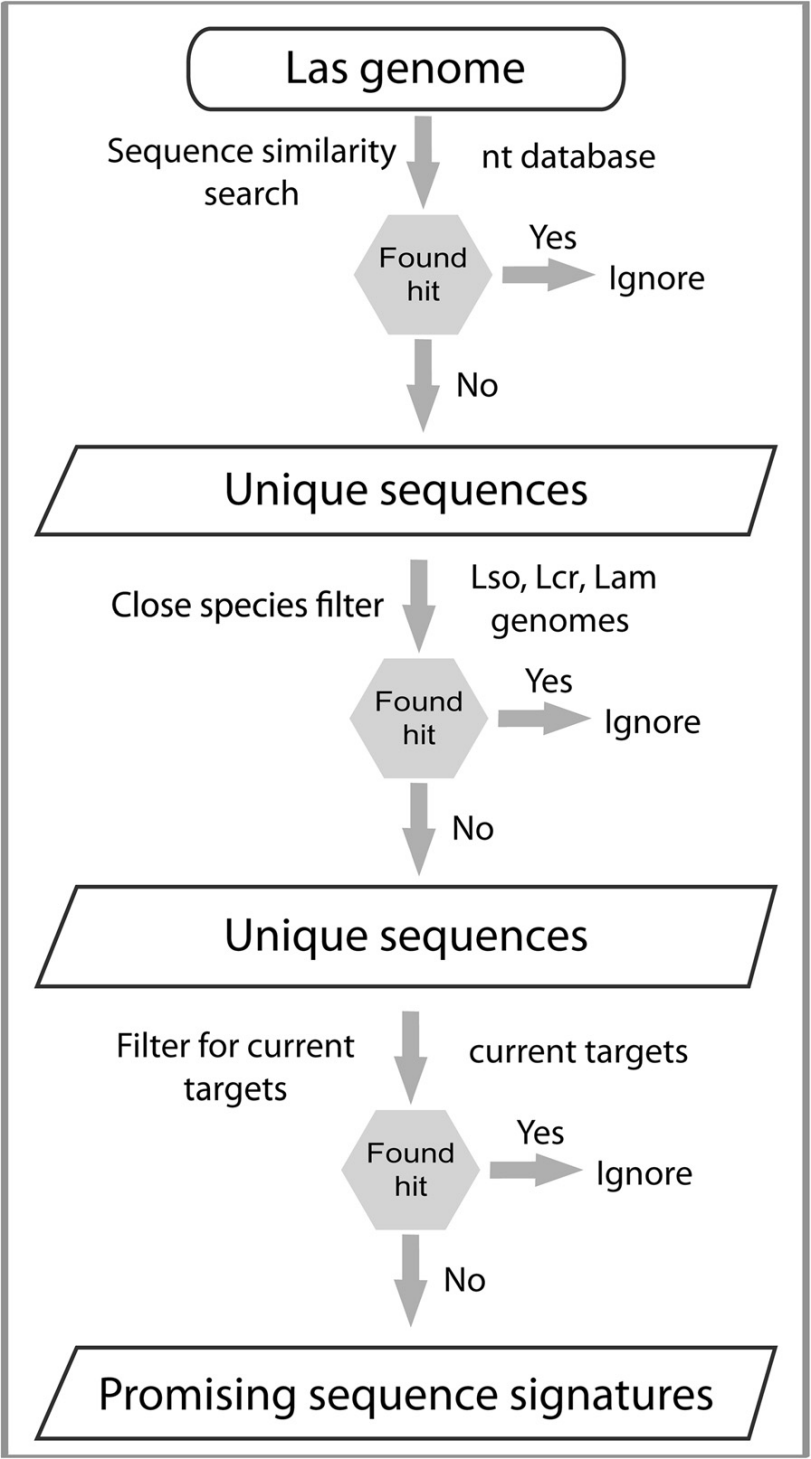
**Pictorial representation of the bioinformatics strategy employed to churn out the unique genic regions from Las genome.** The input and output of each step are shown in oval or square boxes. Actions taken are noted to the left side of the arrow mark, while the information used is indicated to the right side of the arrow.

There are ~1100 genes assigned to the Las genome. Therefore, manual searching of each of these sequences against the nt database using BLAST program [42,43] is a laborious and time consuming procedure. Hence, we automated this sequence similarity search step by developing a standalone PERL script (Additional file 1). This script performed the similarity searches for each of the Las gene against the specified database with hard-coded parameters for the BLAST program. Further, manual analysis of the resulting BLAST search output files is also laborious and time consuming; we therefore, automated this step by developing a second PERL script (Additional file 2). This script automatically parsed all the BLAST output files and returned the Las sequences for which, no hits were found in other organisms. We refer to these sequences as probable unique sequences, because there are nearly no identical sequences found in other organisms (Figure 1).

We performed the sequence similarity searches first by using stringent E-value of ≤ 1 × 10^-3^ against nt database (Figure 1). This search resulted in ~200 sequences that are unique to Las. This set of sequences is relatively high to validate experimentally; therefore, to further reduce the number of unique sequences, we performed the second sequence similarity search with a relaxed E-value of ≤ 1. This search resulted in 38 unique sequences. The E-value of ≤ 1 excludes the sequences with even little similarity to other organisms. Therefore, the resulting 38 unique sequences are considered unique to Las and constitute the promising candidates for qRT-PCR based detection (Figure 1).

We further searched the 38 unique sequences of Las against the phylogenetically closely related Lso, Lam, and Lcr. Because these organisms are closely related, we used the stringent E-value threshold of ≤ 1 × 10^-3^ for this similarity search. In order to achieve this E-value, the sequences need to be highly similar between the Las, Lso, Lam, and Lcr. Therefore, this close species filter procedure potentially eliminates all the Las sequence targets that could lead to false positive results in qRT-PCR based molecular diagnostic assays. Consequently, we further eliminated four conserved sequences from the list of 38 unique sequences, resulting in a total of 34 potential sequence signatures. We could not apply this close species filter step against Laf genome as its genome is yet to be sequenced.

Five (~15%) of the 34 unique gene sequences namely CLIBASIA_05545, CLIBASIA_05555, CLIBASIA_05560, CLIBASIA_05575 and CLIBASIA_05605 are in the prophage region of the Las genome. All these five unique sequences are located upstream of the genomic locus CLIBASIA_05610 encoding a phage terminase. There are possibly 30 genes that represent the complete prophage genome within the Las genome [25,44], of which 16 open reading frames (ORFs) are upstream of the phage terminase, while the remaining 13 ORFs are downstream. The prophage genes CLIBASIA_05610 (primer pair 766 F and 766R) and CLIBASIA_05538 (primer pair LJ900F and LJ900R) have been targeted in previous studies by both conventional as well as qRT-PCR based assays [25,44].

We further analyzed the genomic orientation of the 34 unique genes. This analysis revealed that 15 (~44%) of them are oriented on the sense strand, while the remaining 19 (~56%) were present on the anti-sense strand (Additional file 3: Figure S1). The sequence length of these unique genes ranged from 93 to 2595 base pairs (bp) (Additional file 4: Table S1).

### Designing of Las specific primers and experimental validation of the specificity and sensitivity of qRT-PCR assay to detect Las

Based on the genome sequence of Las strain psy62, we designed 34 qRT-PCR primer pairs that specifically target the 34 unique sequences identified in our bioinformatic analyses (Additional file 4: Table S1). We designed the melting temperature (Tm) of each of these primers to range from 59°C to 65°C with an optimum of 62°C. The GC content of the primers ranged from 35% to 65% with an optimum of 50%. The PCR amplicon sizes for each primer set are between 84 to 185 bp (Additional file 4: Table S1).

In addition to the novel primers designed in this work, we also used a set of control primers that have been previously used in a qRT-PCR based detection of Las. These known primers include 16S rDNA pairs specific to the three different *Candidatus* Liberibacter species (HLBasf/r: Las, HLBamf/r: Lam and HLBaf/r: Laf) [23], β-operon (CQULA04f/r: β-operon) [26], intragenic repeats regions of the prophage sequence (LJ900f/r: Prophage) [25], and the primer pair specific to the plant cytochrome oxidase (COXf/r: COX) gene [23] as a positive endogenous control.

We performed qRT-PCR assays to test the specificity of the designed primers using total DNA extracted from Las-infected citrus plants as a template. To further validate the specificity of these primers, we also included total DNA from the phylogenetically closely related species Lam and Laf in our test. Additionally, DNA extracted from healthy citrus plant was used as a negative control, whereas water served as a no template control. The results of qRT-PCR assays are listed in Table 1.

**Table 1:** Specificity and sensitivity of the novel primers in the detection of Las as shown by qRT-PCR assay

**Primer pairs**	**Target gene**	**Las**			**C**_**T **_**value of the qRT-PCR**^**#**^									
					**Negative control**				**Other controls**					
		**C**_**T **_**value**	***R***^**2 **^**value**^**†**^	**Slope**^**†**^	**Laf**	**Lam**	**Healthy plant tissue**	**Water**	**C1**	**C2**	**C3**	**C4**	**C5**	**C6**
P1	CLIBASIA_05555	20.54	0.9944	-0.2883	UD	UD	UD	UD	UD	UD	UD	UD	UD	UD
P2	CLIBASIA_04315	19.99	0.9867	-0.2849	UD	UD	UD	UD	UD	UD	UD	UD	UD	UD
P3	CLIBASIA_05575	20.15	0.9991	-0.2847	UD	UD	UD	UD	UD	UD	UD	UD	UD	UD
P4	CLIBASIA_05465	19.52	0.9618	-0.2897	UD	UD	UD	UD	UD	UD	UD	UD	UD	UD
P5	CLIBASIA_01460	19.48	0.9995	-0.2969	UD	UD	UD	UD	UD	UD	UD	UD	UD	UD
P6	CLIBASIA_05145	22.29	0.9971	-0.3057	UD	UD	UD	UD	UD	UD	UD	UD	UD	UD
P7	CLIBASIA_05545	20.11	0.9972	-0.3407	UD	UD	UD	UD	UD	UD	UD	UD	UD	UD
P8	CLIBASIA_05560	19.92	0.9982	-0.3132	UD	UD	UD	UD	UD	UD	UD	UD	UD	UD
P9	CLIBASIA_02025	20.12	0.9875	-0.2743	UD	UD	UD	UD	UD	UD	UD	UD	UD	UD
P10	CLIBASIA_05605	20.18	0.9945	-0.2781	UD	UD	UD	UD	UD	UD	UD	UD	UD	UD
P11	CLIBASIA_03090	23.61	0.9997	-0.2867	UD	UD	UD	UD	UD	UD	UD	UD	UD	UD
P12	CLIBASIA_03875	27.47	0.9992	-0.2563	UD	UD	UD	UD	UD	UD	UD	UD	UD	UD
P13	CLIBASIA_02305	UD	NT	NT	UD	UD	UD	UD	UD	UD	UD	UD	UD	UD
P14	CLIBASIA_05495	21.25	0.9974	-0.2594	UD	UD	UD	UD	UD	UD	UD	UD	UD	UD
P15	CLIBASIA_02660	UD	NT	NT	UD	UD	UD	UD	UD	UD	UD	UD	UD	UD
P16	CLIBASIA_02715	20.26	0.9411	-0.3480	UD	UD	UD	UD	UD	UD	UD	UD	UD	UD
P17	CLIBASIA_03110	20.11	0.9994	-0.2786	UD	UD	UD	UD	UD	UD	UD	UD	UD	UD
P18	CLIBASIA_03675	20.02	0.9967	-0.2780	UD	UD	UD	UD	UD	UD	UD	UD	UD	UD
P19	CLIBASIA_03725	19.91	NT	NT	35.29	UD	UD	UD	UD	UD	UD	UD	UD	UD
P20	CLIBASIA_03955	21.08	NT	NT	UD	UD	UD	UD	37.41	UD	UD	UD	UD	UD
P21	CLIBASIA_04030	20.30	NT	NT	UD	UD	UD	UD	32.93	UD	UD	UD	UD	UD
P22	CLIBASIA_04150	24.00	NT	NT	UD	UD	UD	UD	UD	UD	UD	UD	UD	UD
P23	CLIBASIA_04310	20.76	0.991	-0.2976	UD	UD	UD	UD	UD	UD	UD	UD	UD	UD
P24	CLIBASIA_04330	20.85	0.9986	-0.2635	UD	UD	UD	UD	UD	UD	UD	UD	UD	UD
P25	CLIBASIA_04405	21.60	0.9987	-0.3051	UD	UD	UD	UD	UD	UD	UD	UD	UD	UD
P26	CLIBASIA_04425	20.41	0.9994	-0.3032	UD	UD	UD	UD	UD	UD	UD	UD	UD	UD
P27	CLIBASIA_02645	21.77	NT	NT	38.61	UD	UD	UD	UD	UD	UD	UD	UD	UD
P28	CLIBASIA_04515	22.00	NT	NT	38.63	UD	UD	UD	UD	UD	UD	UD	UD	UD
P29	CLIBASIA_04530	19.00	0.9919	-0.2852	UD	UD	UD	UD	UD	UD	UD	UD	UD	UD
P30	CLIBASIA_04550	22.48	0.9938	-0.2708	UD	UD	UD	UD	UD	UD	UD	UD	UD	UD
P31	CLIBASIA_05230	21.68	0.9941	-0.2771	UD	UD	UD	UD	UD	UD	UD	UD	UD	UD
P32	CLIBASIA_05480	21.48	0.988	-0.2776	UD	UD	UD	UD	UD	UD	UD	UD	UD	UD
P33	CLIBASIA_04475	20.84	0.9913	-0.2644	UD	UD	UD	UD	NT	UD	UD	UD	UD	UD
P34	CLIBASIA_05505	22.70	0.9893	-0.2791	UD	UD	UD	UD	NT	UD	UD	UD	UD	UD
CQULA04F/R	β-operon	22.11	NT	NT	UD	UD	UD	UD	NT	NT	NT	NT	NT	NT
LJ900f/r	Prophage	22.25	NT	NT	UD	UD	UD	UD	NT	NT	NT	NT	NT	NT
HLBas/r	16Sas	24.33	0.9998	-0.3057	NT	NT	UD	UD	NT	NT	NT	NT	NT	NT
HLBam/r	16Sam	NT	NT	NT	NT	24.68	UD	UD	NT	NT	NT	NT	NT	NT
HLBaf/r	16Saf	NT	NT	NT	21.28	NT	UD	UD	NT	NT	NT	NT	NT	NT
COXf/r	Cox	14.80	NT	NT	15.21	18.54	16.15	UD	NT	NT	NT	NT	NT	NT

^†^Las-infected psyllids total DNA was serially diluted spanning up to five logs and used as a template in the qRT-PCR assay. R^2^ and slope were further calculated from a plot of C_T_ values versus log dilution factor. ^#^qRT-PCR was conducted by using template DNA samples of Las, Laf, Lam, C1: *Colletotrichum acutatum* KLA-207, C2: *Elsinoe fawcettii*, C3: *Xanthomonas axonopodis* pv. *citrumelo*1381, C4: *Xanthomonas citri* subsp. *citri* A^w^, C5: *Xanthomonas citri* subsp. *citri* A^*^, C6: *Xanthomonas citri* subsp*. citri* 306. The C_T_ values are average of three replicates for each primer pair. UD: undetected; NT: Not tested.

Most of our novel custom designed primer pairs targeting the unique gene sequences were indeed found to be highly specific to Las, as assessed by qRT-PCR assays (Table 1). Among the 34 primer pairs, 29 produced amplicons only when Las-infected citrus plant DNA was used as a template, with an average C_T_ value ranged from 19.48 to 27.47. Two primer pairs, P13 and P15, didn’t produce any amplicons under the standard conditions tested. The other three primer pairs, P19, P27 and P28, produced amplicons when Las or Laf infected plant DNA was used as a template, indicating P19, P27 and P28 could be used to detect both Las and Laf. We were unable to filter for cross-reactivity of P19, P27 and P28 in the bioinformatic analysis, because the Laf genome sequence is currently unavailable. With the exception of these three primer sets that showed amplicons with Laf template, none of the other primer sets produced any amplicons with DNA of Lam, Laf, and healthy citrus or water as template, which further confirms the specificity of these primers to the Las.

We further evaluated the specificity of these primer sets using DNA templates from various citrus associated fungal and bacterial pathogens including *Colletotrichum acutatum* KLA-207, *Elsinoe fawcettii*, *Xanthomonas axonopodis* pv. *citrumelo *1381, *X. citri* subsp. *citri* strains 306, A^w^, and A^*^. Only two primers sets, P20 and P21 showed unspecific amplification against template DNA extracted from fungal pathogen *C. acutatum* KLA-207 (Table 1). *C. acutatum* causes citrus blossom blight, post-bloom fruit drop and anthracnose symptoms that are phenotypically distinguishable from citrus HLB. The P20 and P21 were not filtered by the bioinformatic analysis since *C. acutatum* genome sequence was unavailable in the database. Because of the complexity of the natural microbial community and the limited number of sequences available in the current nucleotide sequence database, it is impossible to completely filter out all the potential false positives bioinformatically. However, false positives could be identified experimentally by combining the different sets of primer pairs by a consensus approach [37]. We eliminated these two primer sets from further evaluation in this study.

The melting temperature analysis of the amplicons produced from our novel primer set with Las as a template indicated that amplicons were of a single species. This suggests that there is no off target amplification for our primer pairs on the Las genome. Overall, the experimental validation of the 34 novel primer sets specific to unique targets revealed that 27 (~80%) of these targets are indeed specific to the Las genome (Table 1). This demonstrates the significance of the bioinformatics strategy employed here for identifying the suitable target regions for the detection of the bacteria by qRT-PCR based methods. These 27 novel primer pairs were selected for further characterization.

To test the sensitivity of our designed novel primers, serial dilutions of Las-infected psyllid DNA was used as a template in the qRT-PCR assay. This serial dilution qRT-PCR assay indicated that most of our novel primer pairs were able to detect Las up to 10^4^ dilutions from the initial template DNA concentration, which is comparable to that of the primer set targeting Las 16S rDNA (Table 1). However, lower sensitivity was observed in the case of primer pairs P9, P12, P14 and P22, which were eliminated from further study. The remaining 23 primer pairs were able to detect Las up to 10^4^ dilutions, with a correlation co-efficient (R^2^ >0.94) between the C_T_ values and dilutions (Table 1). This demonstrates the high sensitivity of these 23 primers in the detection of Las.

### qRT-PCR detection of Las from plant and psyllid DNA samples isolated from diverse locations in USA and China

**Figure 2:**
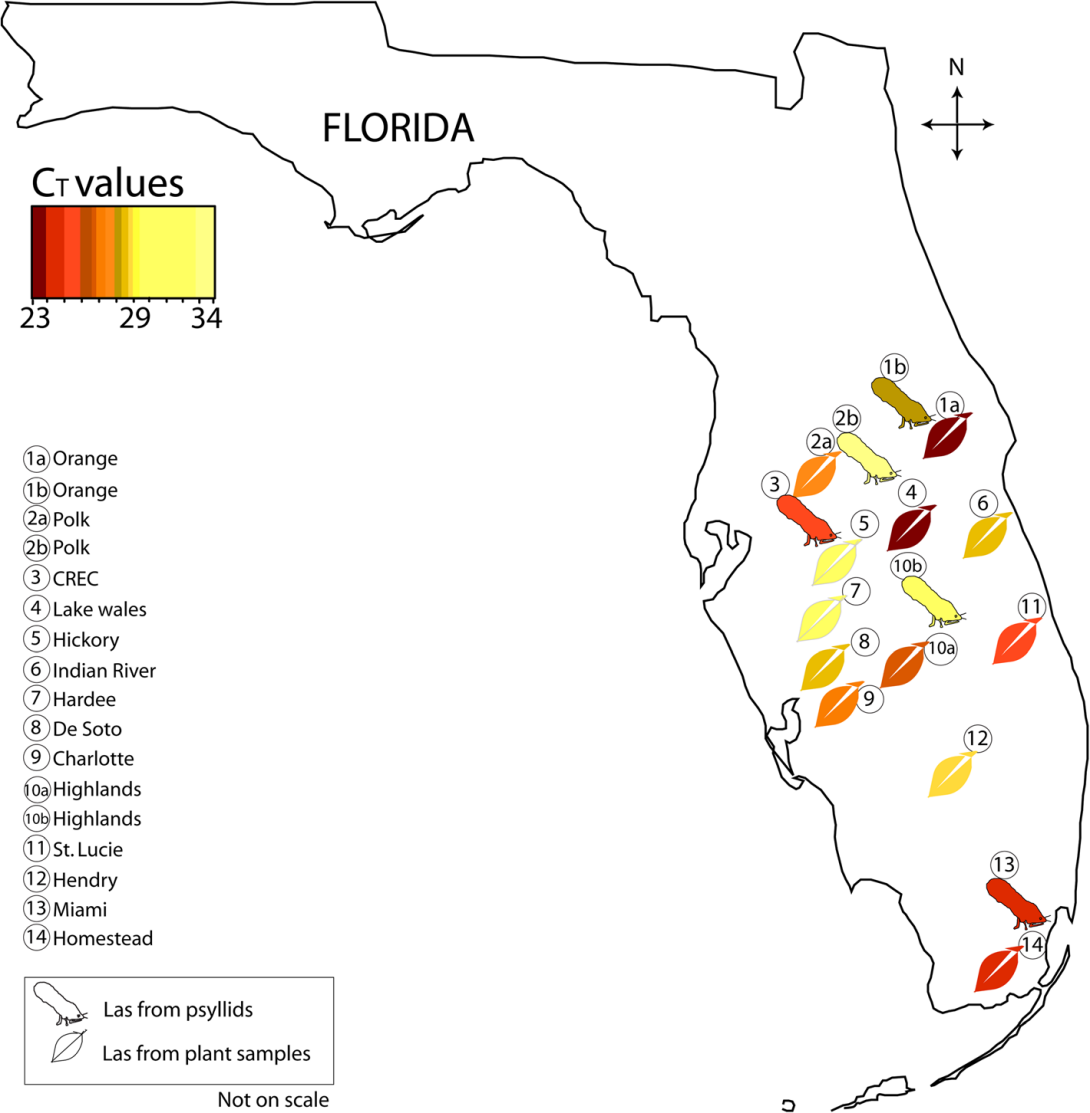
**Schematic representation of the plant and the psyllid samples collected from Florida.** Las-infected plant DNA samples were collected from 12 different locations and psyllids from 5 different locations in Florida, USA. The color shaded symbols for representative plant and psyllid samples are based on their average infection level across all the primer pairs tested based on C_T_ values.

In order to further demonstrate the degree of applicability of the 23 primer pairs in the detection of Las from infected biological material, we performed qRT-PCR on the various Las-infected plant and psyllid DNA samples. Considering the potential variation in nucleotide sequences of Las isolates in different geographic locations that might affect our detection due to the potential nucleotides changes of the target unique genes, we collected Las-infected plant DNA samples as tabulated in Table 2, from not only USA, but also from China, where Las was reported more than 100 years ago [1]. We tested the 23 primer pairs on 17 Las-infected plant DNA samples. Of these 17, 12 were collected from different locations in Florida, USA (Figure 2, Table 2), and the remaining five were collected from different locations in China (Table 2). Additionally, Las-infected psyllid DNA samples collected from five different locations in Florida, USA, were also included in the qRT-PCR assays (Table 3, Figure 2).

**Table 2:** qRT-PCR detection of Las from plant samples that were collected from different locations in USA and China

**Primer pairs**	**C**_**T **_**value of qRT-PCR using infected plant DNA samples as template**^**#**^																
	**DNA samples from Florida, USA**												**DNA samples from China**				
	**Home stead**	**Orange**	**Polk**	**Lake wales**	**Highlands**	**de Soto**	**St Lucie**	**Hendry**	**Hickory**	**Hardee**	**Charlotte**	**Indian river**	**Hai nan**	**Jiang xi**	**Guang xi**	**Yun nan**	**Guang dong**
P1	23.46	22.24	25.33	22.35	24.72	26.35	23.84	26.00	28.89	26.88	24.71	23.73	27.28	UD	32.55	28.18	UD
P2	24.80	23.10	27.41	23.07	26.90	28.31	25.30	29.27	29.90	29.70	26.99	28.94	28.15	25.69	30.68	28.05	27.67
P3	23.97	22.56	25.03	22.64	24.48	26.06	24.11	25.72	28.62	27.99	24.94	24.31	27.11	UD	34.59	29.95	36.57
P4	24.99	23.03	27.71	23.07	27.12	28.30	25.29	28.49	29.03	27.64	27.46	28.12	28.27	25.77	31.48	27.91	28.03
P5	24.44	22.50	27.40	22.47	26.07	28.17	24.45	28.60	28.91	28.53	26.66	27.69	27.31	25.02	31.68	28.49	26.98
P6	25.49	23.16	28.02	23.26	27.14	29.03	25.27	28.84	29.70	30.08	27.53	28.79	27.68	25.26	33.54	27.79	29.30
P7	24.33	23.01	25.30	22.75	25.31	26.03	24.55	26.55	28.16	28.32	24.87	25.07	27.69	UD	34.71	30.97	UD
P8	23.85	22.73	25.80	22.64	24.62	26.00	23.84	26.20	27.66	26.14	25.58	24.20	27.47	UD	31.19	27.40	UD
P10	24.75	23.76	25.96	23.68	26.05	27.38	25.28	27.85	29.09	28.81	26.11	25.43	28.40	UD	31.74	30.97	UD
P11	25.89	24.02	28.51	24.84	28.55	30.52	26.60	30.52	31.72	30.66	28.08	30.54	28.47	26.09	37.56	35.41	29.28
P16	25.50	23.36	27.87	23.20	26.85	28.41	25.67	29.18	29.41	29.54	27.57	28.88	28.10	25.82	30.54	27.27	27.81
P17	25.95	24.09	28.18	23.65	27.54	29.36	26.61	29.90	29.50	31.09	28.14	30.92	29.34	27.01	36.12	30.28	29.20
P18	25.17	23.11	28.02	23.07	27.43	28.75	25.99	28.96	29.36	29.15	28.19	29.09	28.67	26.41	32.17	27.89	28.79
P23	26.41	24.05	29.28	24.35	28.04	30.22	27.75	31.15	32.14	32.95	29.77	31.48	30.31	27.67	36.73	30.86	30.63
P24	26.14	23.83	28.80	23.68	27.58	29.68	27.28	30.86	32.14	31.87	30.71	31.84	29.75	27.51	37.70	30.80	30.05
P25	25.04	22.68	27.97	22.90	26.67	28.28	25.92	28.63	29.04	30.80	27.30	29.77	27.81	25.47	36.49	29.31	29.31
P26	25.11	23.11	27.65	22.86	27.31	28.53	25.71	28.55	29.57	28.66	27.89	29.49	28.41	26.20	31.67	27.50	28.38
P29	24.73	22.72	27.21	22.60	26.65	27.85	25.42	29.36	29.56	29.28	27.17	29.13	27.39	25.33	34.12	28.03	27.51
P30	26.46	24.87	30.59	24.55	28.91	30.73	27.79	29.69	31.25	31.89	28.33	30.69	29.32	26.60	35.91	29.90	30.71
P31	27.19	25.05	29.83	24.77	29.43	31.03	27.88	31.23	32.67	31.14	29.94	30.71	30.28	27.96	34.28	29.94	31.58
P32	26.65	24.65	29.13	23.73	28.24	29.40	25.93	29.44	30.58	30.20	28.11	29.82	28.94	26.60	33.83	29.23	28.77
P33	25.55	23.35	28.08	23.33	27.03	28.42	26.32	30.32	30.58	30.36	27.83	29.79	28.41	25.80	32.99	30.71	28.37
P34	26.49	24.29	29.62	24.46	28.14	29.45	26.22	28.50	29.66	30.85	26.67	29.28	27.24	25.66	36.14	29.07	29.52
HLBas/r	24.76	22.97	27.55	22.80	31.02	29.94	27.24	27.45	28.02	27.20	28.90	27.95	27.06	25.04	30.40	25.93	25.78

^#^Las-infected plant DNA samples were collected from 12 different locations in Florida, USA, and 5 different locations in China. The C_T_ values indicated are average of three replicates for each primer pairs. UD: Under determined.

**Table 3:** qRT-PCR detection of Las from psyllid DNA samples that were collected from different locations in Florida, USA

**Primer pairs**	**C**_**T **_**value of qRT-PCR using infected psyllid DNA samples as template**^**#**^				
	**Polk**	**Miami**	**Highlands**	**Orange**	**CREC**
P1	32.20	24.70	28.76	26.60	24.87
P2	33.64	25.63	29.96	27.71	25.75
P3	32.19	24.39	29.45	26.57	24.95
P4	33.92	25.47	30.09	28.27	25.81
P5	33.12	24.74	28.54	26.22	25.14
P6	33.52	25.45	29.98	27.80	25.60
P7	32.64	27.29	29.36	27.12	25.42
P8	32.46	24.64	28.82	27.48	25.62
P10	33.20	26.30	30.37	28.65	26.52
P11	34.30	26.47	30.34	28.16	26.14
P16	33.76	24.99	28.97	28.23	26.05
P17	34.87	26.08	30.30	28.45	26.91
P18	34.02	25.40	29.73	28.28	26.38
P23	34.69	25.46	30.43	28.60	26.30
P24	34.84	25.58	30.61	28.71	26.45
P25	33.15	24.10	28.46	26.78	24.77
P26	33.40	25.59	29.74	28.07	25.58
P29	33.42	25.14	29.49	27.73	25.29
P30	36.28	26.53	32.12	29.65	27.07
P31	36.10	27.13	31.67	29.94	27.43
P32	35.53	26.40	31.06	29.22	27.23
P33	33.86	25.01	30.00	27.92	25.65
P34	34.99	25.74	30.93	28.58	26.43
HLBas/r	33.41	25.10	29.09	27.86	25.57

^#^Las-infected psyllid DNA samples were collected from 5 different locations in Florida, USA. The C_T_ values indicated are average of three replicates for each primer pair.

All the 23 primer pairs detected Las from all 12 Florida HLB diseased plant samples (Table 2) and 5 psyllid DNA samples (Table 3) in a qRT-PCR assay, which further validated the detection applicability of our novel primers (Figure 2). However, 4 of the 23 primer pairs (P1, P7, P8 and P10) failed to produce amplicons with the infected plant DNA sample from Jiangxi and Guangdong Province, China (Table 2). Primer pair P3 produced no amplicon with Jiangxi sample, and produced unspecific amplicon with the Guangdong sample (with an altered PCR product size, *data not shown*). Interestingly, all these 5 primer pairs target the genes located in prophage region of the Las genome (Additional file 3). These primers (P1, P3, P7, P8 and P10) based on prophage genes could detect Las from Florida, but not from Jiangxi and Guangdong province, China. This is consistent with previous report [44], that prophage was detected in only 15.8% of the 120 HLB diseased citrus samples acquired in Guangdong Province, China, but was detected in 97.4% of the 39 Las positive citrus samples acquired in Yunnan Province, China. This suggests that those prophage genes are not universally present in all strains of Las. Alternately, the prophage sequences were found to be highly variable among the strains tested.

## Conclusions

We have successfully designed 18 novel primer pairs, which are specific to Las. These primers will provide an additional arsenal to qRT-PCR based detection of Las-infected plants and psyllids. Compared to the commonly used primers based on 16S rDNA and β-operon, the 18 primers developed in this study have comparable sensitivity. Moreover, these primers could successfully differentiate Las from Lam, Laf and other common microbes associated with citrus.

## Methods

### Bioinformatics

The nucleotide sequences of Las with accession number NC_012985 [29,45], Lso with accession number NC_014774 [33], Lcr with accession number NC_019907 and comprehensive nucleotide (nt) database (26^th^ July 2012) were downloaded from the NCBI ftp server (ftp.ncbi.nih.gov). The stand-alone BLAST [42,43] was used to search the Las genes against nt, Lso and Lcr databases using a custom-made PERL script 1 (Additional file 1) by varying the E-value with all other parameters kept to a default value. The output files of the BLAST searches were further parsed using a second custom-made PERL script 2 (Additional file 2).

### Plant and psyllid materials and extraction of DNA

Las infected citrus leaf samples with typical visible symptoms were collected from 2 years old infected sweet orange (*Citrus sinensis*) plants maintained at the Citrus Research and Education Center (CREC), Lake Alfred, Florida, USA. As a negative control, the leaves from healthy citrus plants were collected from pathogen-free seedlings grown in the healthy plant greenhouse maintained at CREC, Lake Alfred, Florida, USA. The Laf and Lam infected samples were obtained from South Africa and Brazil respectively. The total DNA from the leaves of citrus was extracted using the protocol mentioned elsewhere [46]. Briefly, the leaves were washed under tap water and surface sterilized in 35% bleach (2% active Chlorine) and 70% (v/v) ethanol for 2 min each. The sterilized leaves were further rinsed three times in sterile water. The midribs from the leaf samples were separated and cut into small pieces. Approximately 100 mg of midrib pieces were used from each sample to extract the DNA using the Wizard® genomics DNA purification kit (Promega, Madison, WI, USA). The extracted DNA was suspended in 100 μl H_2_O.

Las infected psyllids (*Diaphorina citri*) were maintained on confirmed Las-infected sweet orange plants at the CREC, Lake Alfred, FL, USA. In this work, 16 psyllids (around 20 mg) were pooled and the total DNA was extracted using a DNeasy Blood & Tissue Kit (Qiagen, Valencia, CA). The extracted DNA was suspended in 100 μl H_2_O. The quality and quantity of the extracted DNA was determined using a NanoDrop™ 1000 spectrophotometer (NanoDrop Technologies, Inc., Wilmington, DE).

### Quantitative real-time polymerase chain reaction (qRT-PCR)

Gene specific primers were designed using PrimerQuestSM from Integrated DNA technologies (IDT), Coralville, Iowa (Additional file 4: Table S1). qRT-PCR experiments were performed using ABI PRISM 7500 FAST Real-time PCR System (Applied Biosystems, Foster City, CA, US) in a 96-well plate by using an absolute quantification protocol. The reaction mixture in each well contained 12.5 μL 2x FAST SYBR® Green PCR Master Mix reagent (Applied Biosystems), 2 μL DNA template (~30 ng), 0.625 μL of 10 μM of each gene-specific primer pair in a final volume of 25 μL. The standard thermal profile for all amplifications was followed, which involved 95°C for 20 min followed by 40 cycles of 95 °C for 3 sec, and 50°C for 30 sec. All assays were performed in triplicates.

Melting curve analysis was performed using ABI PRISM 7500 FAST Real-time PCR System Software version SDS v1.4 21 CFR Part 11 Module (Applied Biosystems®) to characterize the amplicons produced in a PCR reaction.

## Competing interests

We declare no competing interests.

## Authors’ contributions

NW conceived and coordinated the work and wrote the manuscript. SK designed, performed bioinformatic analysis and wrote the manuscript. SK, QY and NR performed qRT-PCR experiments. SK, QY, XD, CR, TE, MR, MI, GP, and CR participated in experimental design, manuscript writing and provided reagents. All authors read and approved the final manuscript.

## Supplementary Material

Additional file 1 **PERL script 1 facilitates the similarity search in an automated fashion.** This script performs similarity searches against the specified nucleotide sequence database using a stand-alone BLAST program for each of the input gene sequences from the Las genome. Click here for file

Additional file 2 **PERL script 2 facilitates the identification of unique genes to Las.** This script facilitates the identification of unique genes by automatically parsing all the BLAST output files generated from the Additional file 1 PERL script 1and returns the unique gene sequences with no similarity to the DNA sequences of other organisms. Click here for file

Additional file 3: Figure S1 Snapshot of the unique genes identified by bioinformatics is shown in the context of the whole genome of Las. The absolute positions of the regions are shown. The novel unique regions of Las identified in this study are shown in bluish green, while the currently known targets are colored in green. Click here for file

Additional file 4: Table S1 Custom designed primer pairs specific to the unique sequences of Las identified by bioinformatic analysis. The forward and reverse primer pair for each of the unique genic regions is given. The product size for each of the primers is shown along with the %GC content. Click here for file
