# Supplementary figures and images for: Repertoire of novel sequence signatures for the detection of Candidatus Liberibacter asiaticus by quantitative real-time PCR
Source: BMC Microbiol. 2014 Feb 17;14:39. doi: 10.1186/1471-2180-14-39 (PMC4015361; doi:10.1186/1471-2180-14-39)

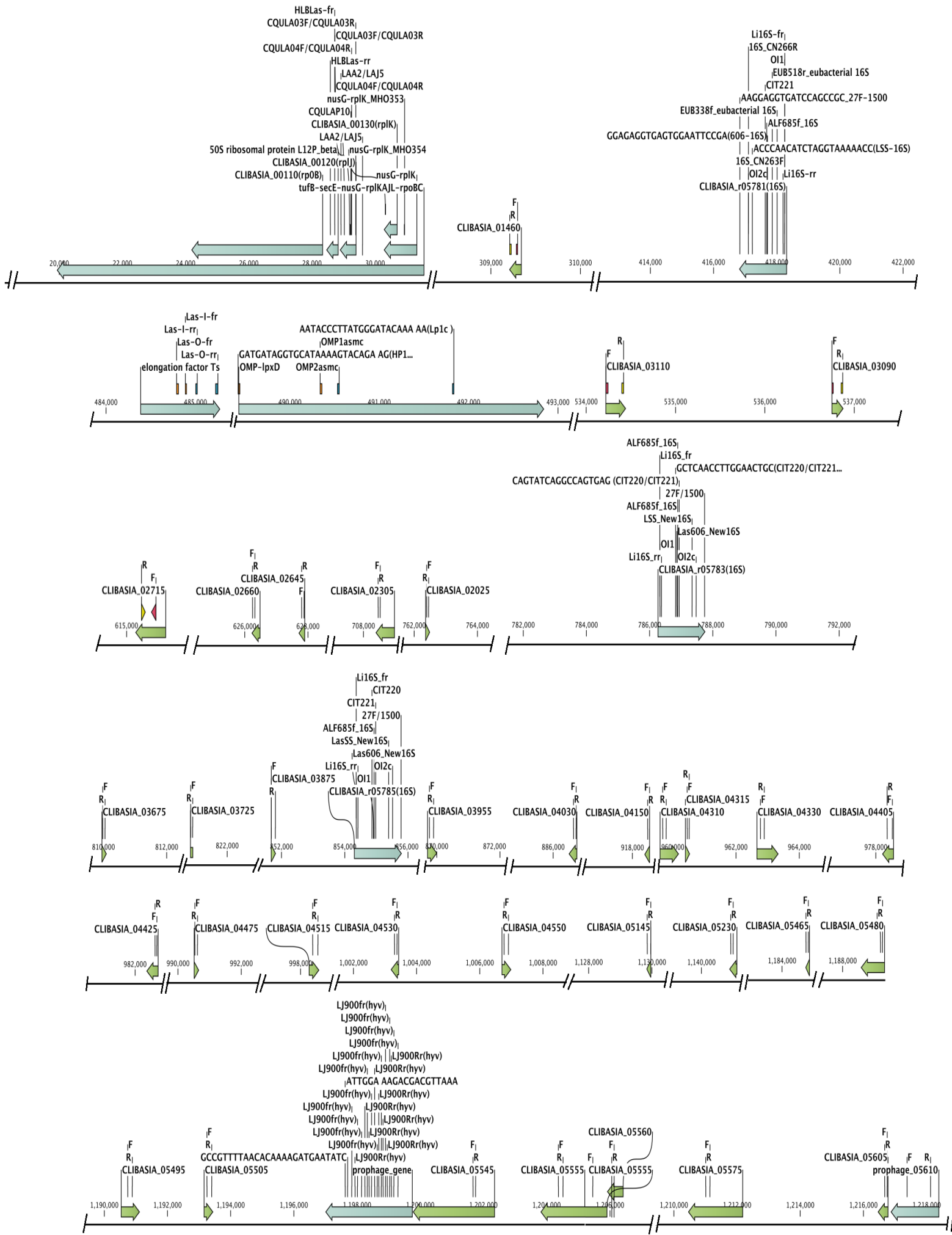

Supplement: Additional file 3: Figure S1 — Snapshot of the unique genes identified by bioinformatics is shown in the context of the whole genome of Las. The absolute positions of the regions are shown. The novel unique regions of Las identified in this study are shown in bluish green, while the currently known targets are colored in green. [file 1471-2180-14-39-S3.pdf]
