# Supplementary material for: Repertoire of novel sequence signatures for the detection of Candidatus Liberibacter asiaticus by quantitative real-time PCR
Source: BMC Microbiol. 2014 Feb 17;14:39. doi: 10.1186/1471-2180-14-39 (PMC4015361; doi:10.1186/1471-2180-14-39)
Supplement: Additional file 4: Table S1 — Custom designed primer pairs specific to the unique sequences of Las identified by bioinformatic analysis. The forward and reverse primer pair for each of the unique genic regions is given. The product size for each of the primers is shown along with the %GC content. [file 1471-2180-14-39-S4.doc]

**Table S1. Custom designed primer pairs specific to the unique sequences of Las identified by bioinformatic analysis.**

| **Primer pairs** | **Target gene locus#** | **PCR product** | | **Primer sequence (5’ to 3’)** | |
| --- | --- | --- | --- | --- | --- |
|  |  | **Size (bp)** | **GC content %** | **Forward primer (f)** | **Reverse primer (r)** |
| P1 | CLIBASIA_05555 | 157 | 43.31 | TTGAATATGACGGTGGGTCTCGCA | ACTTCTCTTTGGCCTCACTGAGCA |
| P2 | CLIBASIA_04315 | 86 | 38.3 | GGGTATAAGCACTTAGGCTTTAAGAAACC | TCACGTCGTAAAGACATCCTGCCT |
| P3 | CLIBASIA_05575 | 156 | 44.87 | TCTGGCAGTAAGGGCGATGAACTT | ATGCATCCAATGAATGGTGCTCGC |
| P4 | CLIBASIA_05465 | 95 | 41.05 | ATGCGAATGGGATAGGAGCGGTTA | AGCCACACTACTTCGTTGACCCAA |
| P5 | CLIBASIA_01460 | 96 | 42.7 | AGGCCTTCATCGCACTGAATCT | CATGCGACTGTGATCCCATAATCC |
| P6 | CLIBASIA_05145 | 133 | 35.33 | TGAAGAGTTTAGTTCGCGACTACTT | CCTCTACCCACATTAGCTTTGTGCT |
| P7 | CLIBASIA_05545 | 104 | 45.19 | TGAGTGTTGATAAGCTCCGTGCGA | AAGCGGTGTTCCGTCGATATCCTT |
| P8 | CLIBASIA_05560 | 96 | 51.04 | ACACCCGCAACGCCTATAAAGGTA | TAGCTCTGTGCCATCCTGCTTCTT |
| P9 | CLIBASIA_02025 | 83 | 48.19 | TCTGTAGCCTGTACATCGGAAACG | AAACTGACGCCTCGAGAAGCAGCAA |
| P10 | CLIBASIA_05605 | 100 | 49 | TTCGGTTCATCGCTATCCAGTGGT | TATATAAACCTGCTGCTCCGCCCA |
| P11 | CLIBASIA_03090 | 128 | 33.59 | ATGCAACGACTTATCGAGATTC | TTGACCAATGCTATTGGAATATCTC |
| P12 | CLIBASIA_03875 | 150 | 31.33 | GGGTTTCGTTTTTGGGTATCAC | CTTACAAAAAAAGGTCATTTGAAAATC |
| P13 | CLIBASIA_02305 | 86 | 50 | AGCCAAGCAGTCTAGGGACGATTT | TTGCCAACATCAACATATCGGCGG |
| P14 | CLIBASIA_05495 | 164 | 43.29 | ATGAAGCGTACTGGCCGATATCCA | TCCGTAACCTCTTGAACGTGACGA |
| P15 | CLIBASIA_02660 | 97 | 43.29 | CGAAAGTTATCTGAGAAGGAGAGC | TCAGGTATGCACCTGTGCTTGTAG |
| P16 | CLIBASIA_02715 | 85 | 43.52 | GTCGATGAATTAAACTCGATATCCTCTCG | CATGAGGATGCACGACTAGATCAGA |
| P17 | CLIBASIA_03110 | 87 | 45.97 | TGCTGTAGCTATTATTGCCGCCGT | TGGTGAGACATCTGGTTTGCGACT |
| P18 | CLIBASIA_03675 | 93 | 43.01 | GGGAAAGATTTCACACGGTATCGG | AGGGCTATACGGACTACAACCCTA |
| P19 | CLIBASIA_03725 | 84 | 48.8 | ACAGGATGTGAACAGGAAGTAGGG | GCGATCTTTCCAGTCACTAAATTGACCC |
| P20 | CLIBASIA_03955 | 152 | 36.84 | CAATTGCAACAACTGCGAGCAACG | ATTCTTTGGCGTGCAGACTTAGCC |
| P21 | CLIBASIA_04030 | 103 | 51.45 | TCCAGTATTTGCAATGGGCACAGC | AAGAGCGACGGGAGCAGGAGGGATA |
| P22 | CLIBASIA_04150 | 80 | 33.75 | ATGGATTACCCTTGTAACAAAGAA | TTCTGTGCTCGCATATCTCCTAGT |
| P23 | CLIBASIA_04310 | 133 | 36.84 | GTCCAATTTATTCCCTCTCACCCG | ACGATCGCTTTAGATCCCGTGGAA |
| P24 | CLIBASIA_04330 | 141 | 40.42 | AGCGGTATGGAAAGGAGATTCGGA | AGATATTGAGCCGCTCGTAACGCT |
| P25 | CLIBASIA_04405 | 187 | 41.17 | CGACTCTTCTTACAATCGGTGGTTG | GCGCGTTCTTCGACTTCTTTCACT |
| P26 | CLIBASIA_04425 | 80 | 46.25 | GTAACGTGCTGAACCTGTATGATGCG | TGCGCACGTTGCTATACGTTCTTC |
| P27 | CLIBASIA_02645 | 97 | 40.2 | AAGTGCTAAGGCTTTACACGAGCG | TTCCAGAAGAGAGCTAGCAATTC |
| P28 | CLIBASIA_04515 | 185 | 30.8 | CAGGCGTAAGTATGTATTAACAGCG | CCCTACGTCCCATAGATACTGATGCT |
| P29 | CLIBASIA_04530 | 101 | 46.53 | TGAGTGGGTGTTCCGAGACTTTAG | TCCTCCGATGGGTTGATCAGCTTT |
| P30 | CLIBASIA_04550 | 162 | 41.97 | CCCATTGGCGTTCTTGATTCTCCA | AGAGCTGAGACGCATATCCTCCAT |
| P31 | CLIBASIA_05230 | 98 | 40.81 | GATGGGACAACGCAACCAATTGAC | TGCGTCGGTTCTTTCCCATTCCTA |
| P32 | CLIBASIA_05480 | 86 | 50 | CACGATGGCGCAAATCGAAGCTAA | TCCTAAAGCACCTAAGGGAAGGGA |
| P33 | CLIBASIA_04475 | 106 | 40.56 | TGCATCGGAGGGATGAGATGGATT | CCCTCACCCATACCGATAAGGATAGGATT |
| P34 | CLIBASIA_05505 | 172 | 42.44 | TCATTAACCAGCGAGGTTGTCCTG | ACTTCCTCCGTAGAACTCAGAACC |

# the target gene locus of each primer pair as indicated in the reference strain *Candidatus* Liberibacter asiaticus psy62.
